# A novel probiotic formula, BLLL, ameliorates chronic stress-induced depression-like behaviors in mice by reducing neuroinflammation and increasing neurotrophic factors

**DOI:** 10.3389/fphar.2024.1398292

**Published:** 2024-07-26

**Authors:** Minxiu Ye, Feng Ji, Chao Huang, Fu Li, Changliang Zhang, Yu Zhang, Runxin Wang, Kai Ma, Xu Lu, Hui Wang

**Affiliations:** ^1^ Department of Pharmacy, Kunshan Hospital of Traditional Chinese Medicine, Kunshan, China; ^2^ Jiangsu Biodep Biotechnology, Jiangyin, China; ^3^ Department of Pharmacology, School of Pharmacy, Nantong University, Nantong, China; ^4^ Department of Pharmacy, Changzhou Geriatric Hospital Affiliated to Soochow University, Changzhou, China; ^5^ Department of Gastroenterology, Henan Provincial People’s Hospital, People’s Hospital of Zhengzhou University, Zhengzhou, Henan, China

**Keywords:** probiotic, depression, neuroinflammation, oxido-nitrosative stress, chronic stress

## Abstract

**Introduction:** Probiotics have been recognized for their various biological activities, including antioxidant and anti-inflammatory properties. This study investigates the therapeutic effect of a novel probiotic formula, BLLL, consisting of *Bifidobacterium breve*, *Lactobacillus plantarum*, *Lactobacillus paracasei*, and *Lactobacillus helveticus*, on chronic stress-induced depression-like behaviors in mice.

**Methods:** The BLLL formula or phosphate-buffered saline (PBS) was given orally at a dose of 2, 4, or 8 × 10^10^ CFU/kg once daily for 10 days in mice treated with chronic unpredictable stress (CUS) treated or vehicle. Depression-like behaviors were assessed using the sucrose preference test (SPT), the forced swimming test (FST), and the tail suspension test (TST). The mRNA and/or protein expression of interleukin-1β (IL-1β), IL-6, tumor necrosis factor-α (TNF-α), IL-4, IL-10, and chitinase-3-like protein 3 (CHI3L1, also known as Ym-1), as well as the concentration of nitrite, malondialdehyde (MDA), glutathione (GSH), and brain-derived neurotrophic factor (BDNF) in the hippocampus and medial prefrontal cortex were examined.

**Results:** The BLLL formula treatment at a dose of 8 × 10^10^ CFU/kg, but not at a dose of 2 or 4 × 10^10^ CFU/kg, improved CUS-induced depression-like behaviors in mice, as shown by the decrease in immobility time in the TST and FST and the increase in sucrose intake in the SPT. Further analysis revealed that BLLL treatment suppressed the CUS-induced increase in IL-1β, IL-6, and TNF-α mRNA and protein levels, as well as the CUS-induced decrease in IL-4, IL-10, and Ym-1 mRNA and/or protein levels in the hippocampus and medial prefrontal cortex. In addition, treatment with the BLLL formula countered the CUS-induced increase in nitrite and MDA levels and the CUS-induced decrease in GSH content and BDNF concentration in the hippocampus and medial prefrontal cortex.

**Conclusion:** These results demonstrate that the novel probiotic formula BLLL ameliorates chronic stress-induced depression-like behavior in mice by suppressing neuroinflammation and oxido-nitrosative stress in the brain.

## Introduction

Chronic harmful stress is a widespread phenomenon in modern society. It can cause various types of mental disorders, including depression (Smail et al., 2020). In the clinic, medications developed according to the monoamine imbalance hypothesis are used to treat depression (Kraus et al., 2017). However, these medications can have numerous harmful effects, such as sleep disturbances (Crawford et al., 2014) and an increased risk of suicide (Moller et al., 2008). Notably, only a small proportion of patients receiving antidepressants experience a significant therapeutic effect (Bollini et al., 1999; Furukawa et al., 2022). Therefore, there is an urgent need to search for new drugs to treat depression triggered by chronic stress.

In recent years, the brain-gut axis has attracted much attention. The gut microbiota has been shown to control brain development, function, and behavior (Sampson and Mazmanian, 2015). Many gastrointestinal diseases are linked to the development of various mental disorders, such as anxiety and depression (Barberio et al., 2021). Further, gut microbiota dysfunction plays a significant role in the progression of mental disorders, including depression (Dinan and Cryan, 2019; Cussotto et al., 2021). Compared to healthy individuals, depression patients tend to have more pro-inflammatory bacteria and fewer anti-inflammatory bacteria in their gut microbiota (Simpson et al., 2021; Liu et al., 2022). Since a systemic inflammatory state is associated with increased gut permeability and can affect the integrity of the brain-blood barrier, the balance of the gut microbiota is closely related to neuroinflammation (Reyes-Martinez et al., 2023).

An enhanced neuroinflammatory response is a new hypothesis to explain the pathogenesis of depression (Ji et al., 2022). Previous studies have consistently observed elevated levels of pro-inflammatory cytokines such as interleukin-1β (IL-1β), IL-6, and tumor necrosis factor-α (TNF-α) in the blood of patients with depression [15–17]. Animal models of depression have confirmed that high concentrations of pro-inflammatory cytokines and low concentrations of anti-inflammatory mediators like IL-10 and IL-4 in the brain correlate with the progression of depression [18–20]. Central infusion of pro-inflammatory cytokines such as IL-1β and interferon-α (INF-α) can induce depression-like behaviors in animals (Anisman et al., 2008; Hayley et al., 2013). Clinically available antidepressants such as fluoxetine have been reported to improve depressive symptoms in part by inhibiting the production of pro-inflammatory cytokines in the brains of chronically stressed mice (Du et al., 2016) or in the blood of patients with depression (Wang et al., 2019). Mechanistic studies have shown that pro-inflammatory cytokines promote the progression of depression by increasing oxido-nitrosative stress (Sulakhiya et al., 2014; Yang et al., 2017) and impairing the expression of brain-derived neurotrophic factor (BDNF) (Calabrese et al., 2014). Therefore, regulating the gut microbiota and shifting the neuroinflammatory response to an anti-inflammatory state while also attenuating oxido-nitrosative stress could mitigate depressive symptoms triggered by harmful stress, which could be a promising strategy for the treatment of depression.

Probiotics are live, nonpathogenic microorganisms that can improve microbial balance and are widely used as dietary supplements and foods (Williams, 2010). Strategies that correct gut microbiota dysfunction with probiotics have been widely reported to alleviate depressive symptoms in animals (Park et al., 2018; Kosuge et al., 2021) and humans (Huang et al., 2016; Tian et al., 2022), which is likely achieved by targeting the nervous system via inhibiting neuroinflammation and oxido-nitrosative stress. However, there are still no clinically available probiotics for the treatment of depression, although numerous probiotics for this purpose are being developed. In the present study, we investigated the effects and mechanisms of a new formula of probiotics called the BLLL formula, consisting of *Bifidobacterium breve*, *Lactobacillus plantarum*, *Lactobacillus paracasei*, and *Lactobacillus helveticus*, provided by Biodep Biotechnology Company, on chronic stress-induced depression-like behaviors in mice. The effects of the individual components of the BLLL formula on depression have been described in prior studies. For example, *Bifidobacterium* has been reported to improve depression-like behaviors and microbiota dysbiosis in animals induced by chronic stress (Tian et al., 2019; Tian et al., 2020). *Lactobacillus plantarum* can alleviate depression-like behaviors in depressed animals by regulating the gut microbiota (Zhu R. et al., 2023) and improving the function of monoamine neurotransmitters and the CREB-BDNF signaling cascade in the hippocampus (Ma et al., 2024). Both *L. paracasei* (Xu et al., 2022) and extracellular vesicles released by *Lactobacillus* paracasei (Kwon et al., 2023) also have antidepressant-like effects in animal models of depression. The role of *L. helveticus* in the regulation of depression has also been described and discussed in numerous studies (Maehata et al., 2019; Partrick et al., 2021). We hypothesize that the BLLL formula can reverse depression-like behavior in chronically stressed mice by altering brain neuroinflammatory responses and oxido-nitrosative stress.

## Materials and methods

### Animals

Seven-week-old male C57BL/6J mice (body weight: 22–26 g) were purchased from Beijing Vital River Laboratory Animal Technology Co, Ltd (Beijing, China) and housed five per cage for 1 week before experimental procedures started. All mice were kept under conditions with a 12-h light-dark cycle (lights on from 07:00 to 19:00), 23°C ± 1°C ambient temperature, 55% ± 10% relative humidity, without any environmental enrichment, and free access to food and water. Animal experiments were approved by the Animal Ethics Committee of Nantong University (Permit Number: 2110836) and conducted according to internationally accepted guidelines for the use of animals in toxicology adopted by the Society of Toxicology in 1999.

## Materials

The BLLL formulation containing *B. breve* (25%), *L. plantarum* (25%), *L. paracasei* (25%), and *L. helveticus* (25%) was provided by Biodep Biotechnology Company and stored at −20°C.

### Procedure for drug treatment, behavioral tests, and biochemical measurements

**FIGURE 1:**
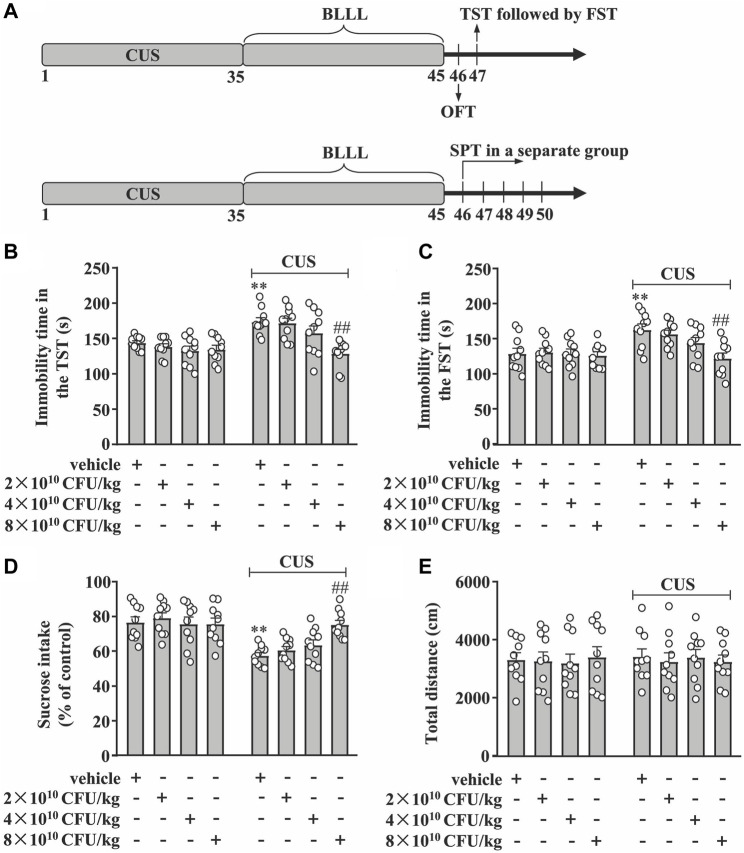
Dose-dependent effect of BLLL formula on CUS-induced depression-like behavior. **(A)** A schematic diagram showing the timeline for the evaluation of the dose-dependent effect of BLLL formula on CUS-induced depression-like behaviors in mice. **(B, C)** Quantitative analysis showing the effect of different doses of BLLL formula treatment (2×10^10^, 4×10^10^, and 8 × 10^10^ CFU/kg; 10 days) on CUS-induced increases in the immobility time in TST **(B)** and FST **(C)** in mice (n = 10, **p < 0.01 vs vehicle; ##*p* < 0.01 vs vehicle + CUS). **(D)** Quantitative analysis showing the effect of different doses of BLLL formula treatment (2×10^10^, 4×10^10^, and 8 × 10^10^ CFU/kg; 10 days) on CUS-induced decrease in sucrose intake in SPT (n = 10, **p < 0.01 vs vehicle; ##*p* < 0.01 vs vehicle + CUS). **(E)** Quantitative analysis showing the effect of different doses of BLLL formula treatment (2×10^10^, 4×10^10^, and 8 × 10^10^ CFU/kg; 10 days) on the total distance of mice treated with vehicle, BLLL formula, and/or CUS (n = 10). Data are shown as mean ± SEM.

Mice exposed or not to chronic unpredictable stress (CUS) were orally administered the BLLL formula at a dose of 2, 4, or 8 × 10^10^ CFU/kg once daily for 10 days. The control animals, exposed or not to CUS, received the same amount of phosphate-buffered saline (PBS). PBS solution is a neutral fluid with minimal chemical reactions with other substances, making it safe and effective. In addition, the concentration of PBS is similar to the cell fluid of mice; therefore, it does not lead to dehydration of the cells. To this end, PBS has been widely used in numerous previous studies as a control substance to explore the advantages of probiotics (Ducray et al., 2020; Mulaw et al., 2020; Sun et al., 2020; Xiao et al., 2022; Fitri et al., 2023). The BLLL formula was dissolved in PBS immediately before administration. Gavage tubes containing BLLL or PBS were administered 200 μL per mouse at 8 a.m. In experiments to assess depression-like behaviors, a separate cohort of stress-naïve and CUS mice was divided into eight groups administered either vehicle or 2, 4, or 8 × 10^10^ CFU/kg BLLL (10 in each group). Behavioral tests were carried out 24 h after the final probiotic treatment. These tests included the sucrose preference test (SPT), the tail suspension test (TST), the forced swimming test (FST), and the open field test (OFT). The OFT was performed 1 day before the TST, followed by the FST. The SPT was conducted on a separate group of mice. The dose of BLLL application was chosen according to previous studies (Gao et al., 2022; Ramalho et al., 2022). The schematic diagrams for the specific arrangement of the tests for depression-like behaviors are shown in Figure 1A. The mice in the control group were kept in the same room where the stressed mice were exposed. The researchers were blinded to group assignment during the experiments and data analysis. The behavioral tests were performed during the light phase; each group consisted of 10 mice. To avoid a possible influence of the behavioral tests on the molecular analysis, we chose a dose of 8 × 10^10^ CFU/kg BLLL, and divided the other animals into four groups (vehicle, BLLL, CUS + vehicle, and CUS + BLLL). Each group consisted of eight mice to prepare the hippocampus and medial prefrontal cortex for further real-time PCR experiments and biochemical measurements. For real-time PCR and biochemical assays, the hippocampal and mPFC tissues were manually dissected bilaterally using a scalpel on wet ice. The mPFC tissues were collected according to “The Mouse Brain Atlas in Stereotaxic Coordinates” by Keith Franklin and George Paxinos (2008, Academic Press) using a mouse brain matrix from Ted Pella, Inc. Since the sample size of the animals is relatively small, the mice were randomly assigned to the experimental groups using the stratified randomization method. All mice were transported in their home cage and temporarily kept in a new cage in the experimental room during the test. The environment in the experimental room was the same as that in the housing room.

### Establishment of the CUS model

The CUS model was established according to previous studies [48–53]. In brief, mice were exposed daily (35 days) over a period of 5 weeks to two of the following stressors in random order: shaking the cage for 1 h, light throughout the night (12 h), housing in a 4°C cold room for 1 h, light restraint (by housing in small cages) for 2 h, tilting the cage 45° for 14 h, no light for 3 h during the daylight phase, wet cage for 14 h, flashing light for 6 h, noise in the room for 3 h, and water deprivation for 12 h during the dark phase. This method is well described as a model for depression and was performed according to a standard protocol.

### Behavioral tests

The TST and the FST were performed following previous studies (Tong et al., 2017; Liu et al., 2021; Wang et al., 2021; Wang Y. et al., 2022; Tang et al., 2022; Wu et al., 2022). Mice were habituated to the test room for 20 min before the session began. In the TST, mice were suspended 50 cm above the floor for 6 min with tape placed approximately 1 cm from the tip of their tails, and were considered immobile when they hung passively and were completely motionless (any mouse that climbed on its tail was excluded from further analysis). For FST, mice were placed in a clear glass cylinder (height 25 cm and diameter 10 cm) filled to 10 cm with water at 25°C ± 1°C for 6 min. They were considered immobile when they floated in the water without struggling, making only the movement necessary to keep their heads above the water. The duration of immobility during the last 4 min of floating in the TST or forced swimming in the FST was recorded with a video (Anhui Zhenghua Biological Instrument Equipment Co. Ltd, Huaibei, China).

The SPT was performed following one of our previous studies (Lu et al., 2022). Mice were given the choice to drink from two bottles in individual cages, one containing 1% sucrose solution and the other containing water, and were acclimated to the two-bottle conditions for 2 days. The position of the two bottles was changed every 6 h to prevent side preference. On the test day, after 24 h of food and water deprivation, the mice were first acclimated to the test room for 20 min, and then exposed to the weighed bottles for 6 h, during which time the position of the bottles was exchanged. Sucrose preference was calculated as the percentage of sucrose solution consumed relative to total fluid intake.

The OFT was performed following one of our previous studies (Li et al., 2020). Mice were acclimated to the test room for 20 min before the session began in a dimly lit environment with a red light bulb (50 W) on the ceiling. During the test, each mouse, initially placed in the center area, was allowed to move freely in a cubic chamber (360 (W)×360 (H)×360 (D) mm) for 5 min. The time spent in the central area and the total distance of the mice in the open field were recorded by the automatic analysis system (Anhui Zhenghua Biological Instrument Equipment Co. Ltd, Huaibei, China). The apparatus in each instrument was thoroughly cleaned with 70% ethanol after each trial to remove olfactory cues.

### Quantitative real-time PCR

24 h after the last probiotic treatment, the mice were killed by rapid decapitation. The hippocampus and medial prefrontal cortex (from the same animals), two brain regions with severe damage associated with depression (Price and Duman, 2020), were immediately harvested. Total RNA in these tissues was extracted using an RNeasy Mini Kit according to the manufacturer’s instructions (Qiagen, GmbH, Hilden, Germany). The first strand of cDNA was generated using a reverse transcription system (Promega, Madison, WI, USA). Real-time PCR was performed using a reaction system containing 1 × Faststart SYBR Green Master Mix (Roche Molecular Biochemicals), 2 μL diluted cDNA, 2 mM MgCl_2_, and 0.5 μM primers. Primers for TNF-α, IL-1β, IL-6, IL-4, IL-10, chitinase-3-like protein 3 (CHI3L1, also named Ym-1), and GAPDH are as follows: TNF-α, 5′-CTG​TGA​AGG​GAA​TGG​GTG​TT-3′ F), 5′-GGTCAC TGTCCCAGCATCTT-3′ (R); IL-β, 5′-TGGAAAAGC GGTTTGTC TTC-3′ F), 5′-TAC​CAG​TTG​GGG​AAC​TCT​GC-3′ (R); IL-6: 5′-AGA​GAT​ACA​AAG​AAA​TGA​TGG​A-3′ (F), 5′-AGC​TAT​GGT​ACT​CCA​CAA​GAC​CA-3′ (R); IL-4, 5′-CAG​CTA​GTT​GTC​ATC​CTG​CTC​TTC-3′ (F), 5′- GCC​GAT​GAT​CTC​TCT​CAA​GTG​A-3′ (R); IL-10, 5′-GGC​AGA​GAA​CCA​TGG​CCC​AGA​A-3′ (F), 5′-AAT​CGA​TGA​CAG​CGC​CTC​AGC​C-3′ (R); Ym-1, 5′-TCA​CTT​ACA​CAC​ATG​AGC​AAG​AC-3′ (F), 5′- CGG​TTC​TGA​GGA​GTA​GAG​ACC​A-3′ (R); GAPDH, 5′-GGCCT TCCGTGTTCCTAC-3′ (F), 5′-TGT​CAT​CAT​ATC​TGG​CAG​GTT-3′ (R). PCR products were detected by observing the increase in the fluorescence intensity of the double-stranded DNA-binding dye SYBR Green. Gene expression analysis was performed using the 2^−ΔΔCt^ method (Livak and Schmittgen, 2001). Samples were assayed in triplicate, and values were normalized to the housekeeping gene GAPDH.

### Detection of BDNF concentrations

BDNF concentrations in the hippocampus and medial prefrontal cortex were measured using the BDNF DuoSet Kit (R&D System; DY248) according to the manufacturer’s protocol. In brief, a 96-well microplate was coated (overnight at room temperature) with 100 μL per well of diluted capture antibody, followed by careful washing on the second day. The plates were then blocked with reagent diluent (300 μL, 1 h at room temperature). Then, 100 μL of the sample or standards in reagent dilution were added to the prepared wells and incubated for 2 h at room temperature. Then, the detection antibody (100 μL), the working dilution of streptavidin-HRP (100 μL), the substrate solution (100 μL), and the stop solution (50 μL) were added to each well in turn. Finally, the optical density of each well was determined using a microplate reader set at 450 nm (Molecular Devices, Sunnyvale, CA, USA).

### Detection of pro-inflammatory and anti-inflammatory cytokine concentrations

The concentrations of IL-1β, IL-6, TNF-α, IL-4, and IL-10 in the hippocampus and medial prefrontal cortex were determined according to the manufacturer’s protocol using the corresponding commercial kits (Proteintech, Wuhan, China). The concentrations of IL-1β, IL-6, TNF-α, IL-4, and IL-10 were expressed as picograms per Gram of tissue (pg/g tissue).

### Detection of nitrite contents

Nitrite levels in the hippocampus and medial prefrontal cortex were measured according to the manufacturer’s protocol (Bi Yuntian Biological Technology Institution, Shanghai, China). In brief, we first diluted the standard sample with tissue lysis buffer containing 50 mM Tris-HCl (pH 7.4), 1 mM EDTA, 100 mM NaCl, 20 mM NaF, and 3 mM Na_3_VO_4_ with 1% NP-40, and then added the samples and reaction solutions to a 96-well microplate in the following order: 150 μL of the samples (tissue supernatant in the lysis buffer), 20 μL of the Griess Reagent, and 130 μL of de-ionized water. Nitrite concentration was determined using a spectrophotometric M2 microplate reader (548 nm, Molecular Devices, Sunnyvale, CA, USA) based on a standard curve (0–100 μM/L) derived from NaNO_2_.

### Detection of malondialdehyde (MDA) and reduced glutathione (GSH) content

The levels of MDA and GSH were determined by the method used in one of our previous studies (Yang et al., 2017) by homogenizing brain tissue in 0.1 M phosphate buffer (pH 7.4) (10% w/v). MDA and GSH levels were expressed as μM/g wet tissue. Brain protein was measured according to the Lowry method (Lowry et al., 1951) and was expressed as μM/mg of protein concentration.

### Statistical analysis

Statistical analyses were performed using Graphpad Prism eight from Graphpad Software, Inc. in CA, USA. Additionally, the Kolmogorov-Smirnov test was used to examine the pattern and distribution of the data; Grubbs’ outlier test was used to further identify and exclude potential outliers in the data. The normality of distribution and sphericity were checked using the Mauchly and Shapiro-Wilk tests. The differences between the means of these data were evaluated using a two-way analysis of variance (ANOVA), and wherever necessary, the Bonferroni *post hoc* test was used to evaluate isolated comparisons. In an experiment, if two factors do not interact in the ANOVA and there are main effects on both factors, the main effects should be determined by averaging the effect of one factor over the levels of the other. This problem can be solved using a *post hoc* t-test, described by Wei *et al.*, to further compare the factors that do not interact (Wei et al., 2012). *p* values *<* 0.05 were considered statistically significant. Data are presented as mean ± standard error of the mean (SEM).

## Results

### BLLL formula reverses CUS-induced depression-like behaviors in mice

First, we evaluated the dose-dependent effect of the BLLL formula on CUS-induced behavioral changes in TST, FST, SPT, and OFT in mice. The experimental procedure is shown in Figure 1A. In the TST, the two-way ANOVA showed significant effects for CUS exposure (F_1,72_ = 20.92, *p <* 0.001), BLLL formula treatment (F_3,72_ = 7.27, *p <* 0.001), and CUS × BLLL formula interaction (F_3,72_ = 3.98, *p <* 0.05) (Figure 1B). For FST, the two-way ANOVA showed significant effects for CUS exposure (F_1,72_ = 13.93, *p <* 0.001), BLLL formula treatment (F_3,72_ = 4.12, *p <* 0.01), and CUS × BLLL formula interaction (F_3,72_ = 2.94, *p <* 0.01) (Figure 1C). Post-hoc analysis revealed that 10-day BLLL formula treatment at a dose of 8 × 10^10^ CFU/kg, but not 2 or 4 × 10^10^ CFU/kg, reversed CUS-induced increase in immobility time in TST (Figure 1B) and FST (Figure 1C) in mice.

In the SPT, the two-way ANOVA showed significant effects for CUS exposure (F_1,72_ = 36.18, *p <* 0.001), BLLL formula treatment (F_3,72_ = 2.83, *p <* 0.05), and the CUS × BLLL formula interaction (F_3,72_ = 4.34, *p <* 0.01) (Figure 1D). Post-hoc analysis showed that 10-day BLLL formula treatment at a dose of 8 × 10^10^ CFU/kg, but not 2 or 4 × 10^10^ CFU/kg, reversed CUS-induced decrease in sucrose intake in mice in SPT (Figure 1D).

To rule out the influence of locomotor activity on mouse behaviors, the total distance of mice in the OFT was evaluated. The two-way ANOVA showed no significant effects for CUS exposure (F_1,72_ = 0.02, *p =* 0.89), BLLL formula treatment (F_3,72_ = 0.05, *p =* 0.99), and the CUS × BLLL formula interaction (F_3,72_ = 0.14, *p =* 0.94) (Figure 1E). Post-hoc analysis revealed that 10-day BLLL formula treatment at a dose of either 2, four or 8 × 10^10^ CFU/kg had not affect the total distance of mice in OFT (Figure 1E).

### BLLL formula reverses CUS-induced enhancement of pro-inflammatory response in the hippocampal and medial prefrontal cortex in mice

**FIGURE 2:**
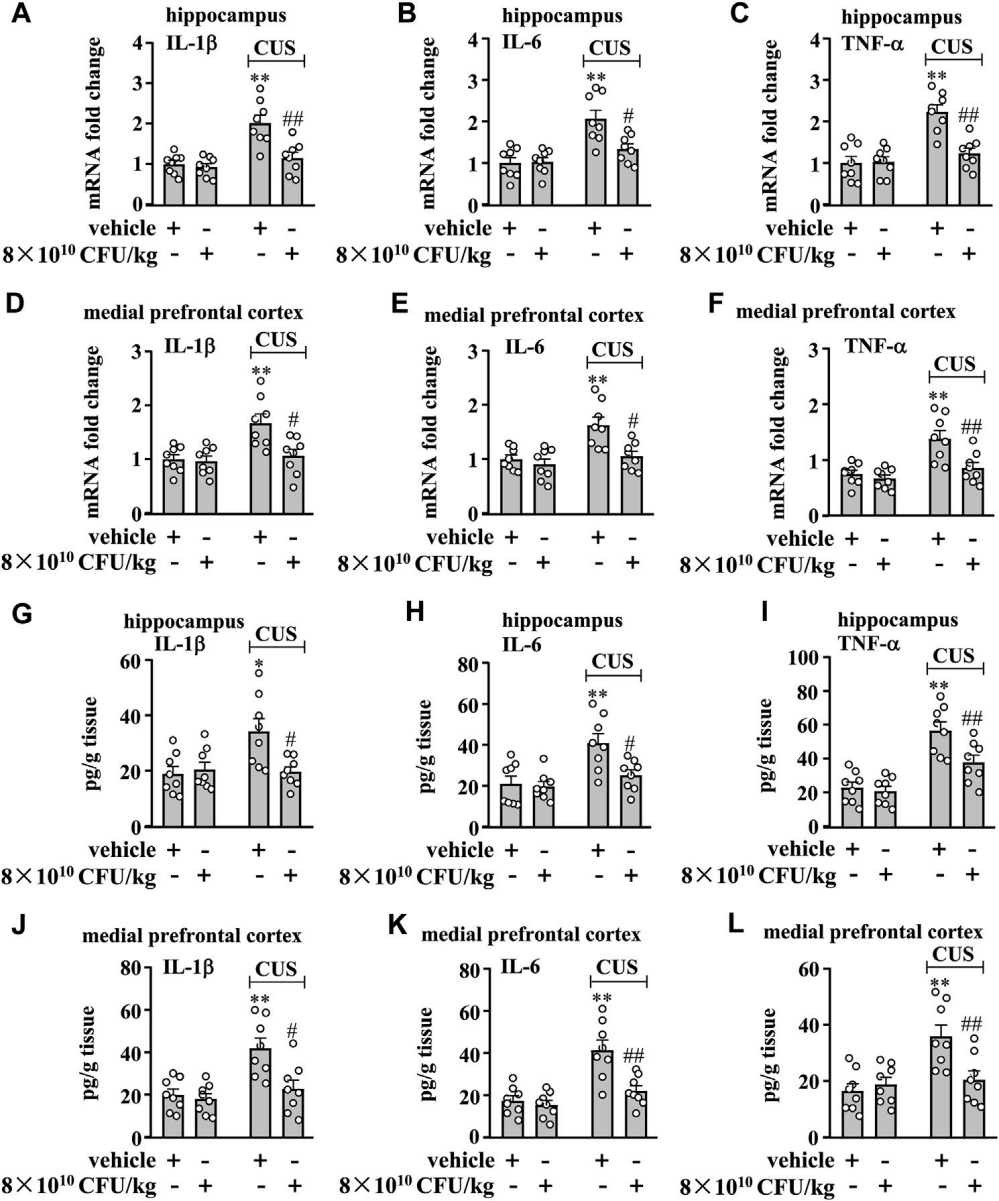
Effect of BLLL formula on pro-inflammatory responses in the brain in chronically stressed mice. **(A–C)** Quantitative analysis shows that 10 days of BLLL formula treatment (8 × 10^10^ CFU/kg) reversed CUS-induced increases in the mRNA expression levels of IL-1β **(A)**, IL-6 **(B)**, and TNF-α **(C)** in the hippocampus in mice (n = 8, **p < 0.01 vs. vehicle; #*p* < 0.05 or ##*p* < 0.01 vs vehicle + CUS). **(D–F)** Quantitative analysis shows that 10 days of BLLL formula treatment (8 × 10^10^ CFU/kg) reversed CUS-induced increases in the mRNA expression levels of IL-1β **(D)**, IL-6 **(E)**, and TNF-α **(F)** in the medial prefrontal cortex in mice (n = 8, **p < 0.01 vs vehicle; #*p* < 0.05 or ##*p* < 0.01 vs. vehicle + CUS). (g–i) Quantitative analysis shows that 10 days of BLLL formula treatment (8 × 10^10^ CFU/kg) reversed CUS-induced increases in levels of IL-1β **(G)**, IL-6 **(H)**, and TNF-α **(I)** in the hippocampus in mice (n = 8, *p < 0.05 or **p < 0.01 vs vehicle; #*p* < 0.05 or ##*p* < 0.01 vs vehicle + CUS). **(J–L)** Quantitative analysis shows that 10 days of BLLL formula treatment (8 × 10^10^ CFU/kg) reversed CUS-induced increases in levels of IL-1β **(J)**, IL-6 **(K)**, and TNF-α (L) in the medial prefrontal cortex in mice (n = 8, **p < 0.01 vs. vehicle; #*p* < 0.05 or ##*p* < 0.01 vs. vehicle + CUS). Data are shown as mean ± SEM.

Next, we evaluated the effect of 10 days of BLLL formula treatment on CUS-induced enhancement of pro-inflammatory responses in the hippocampus and medial prefrontal cortex in mice. To this end, we first evaluated the effect of the BLLL formula on pro-inflammatory cytokine mRNA expression in the hippocampus and medial prefrontal cortex in mice treated with or without the BLLL formula. A two-way ANOVA for levels of IL-1β, IL-6, and TNF-α mRNA in the hippocampus showed significant effects for CUS exposure (IL-1β mRNA: F_1,28_ = 21.33, *p* < 0.001, IL-6 mRNA: F_1,28_ = 22.69, *p* < 0.001, TNF-α mRNA: F_1,28_ = 24.96, *p* < 0.001), BLLL formula treatment (IL-1β mRNA: F_1,28_ = 12.43, *p* < 0.01; IL-6 mRNA: F_1,28_ = 5.82, *p* < 0.05; TNF-α mRNA: F_1,28_ = 11.47, *p* < 0.01), and the CUS × BLLL formula interaction (IL-1β mRNA: F_1,28_ = 8.74, *p* < 0.01; IL-6 mRNA: F_1,28_ = 6.76, *p* < 0.05; TNF-α mRNA: F_1,28_ = 13.03, *p* < 0.01) (Figures 2A–C). Post-hoc analysis revealed that 10-day BLLL formula treatment at a dose of 8 × 10^10^ CFU/kg reversed CUS-induced increases in the expression levels of IL-1β (Figure 2A), IL-6 (Figure 2B), and TNF-α (Figure 2C) mRNA in the hippocampus.

For the levels of IL-1β, IL-6, and TNF-α mRNA in the medial prefrontal cortex, the ANOVA showed significant effects for CUS exposure (IL-1β mRNA: F_1,28_ = 10.38, *p* < 0.01, IL-6 mRNA: F_1,28_ = 13.30, *p* < 0.01, TNF-α mRNA: F_1,28_ = 17.37, *p* < 0.001), BLLL formula treatment (IL-1β mRNA: F_1,28_ = 7.22, *p* < 0.05; IL-6 mRNA: F_1,28_ = 10.01, *p* < 0.01; TNF-α mRNA: F_1,28_ = 9.46, *p* < 0.01), and the CUS × BLLL formula interaction (IL-1β mRNA: F_1,28_ = 5.91, *p* < 0.05; IL-6 mRNA: F_1,28_ = 4.89, *p* < 0.05; TNF-α mRNA: F_1,28_ = 5.13, *p* < 0.05) (Figures 2D–F). Post-hoc analysis revealed that 10-day BLLL formula treatment at a dose of 8 × 10^10^ CFU/kg could reverse CUS-induced increase in the expression levels of IL-1β (Figure 2D), IL-6 (Figure 2E), and TNF-α (Figure 2F) mRNA in the medial prefrontal cortex.

Next, we evaluated the effect of the BLLL formula on pro-inflammatory cytokine protein levels in the hippocampus and medial prefrontal cortex in mice treated with or without the BLLL formula. A two-way ANOVA for levels of IL-1β, IL-6, and TNF-α in the hippocampus showed significant effects for CUS exposure (IL-1β: F_1,28_ = 5.50, *p* < 0.05, IL-6: F_1,28_ = 13.31, *p* < 0.01, TNF-α: F_1,28_ = 40.99, *p* < 0.001), BLLL formula treatment (IL-1β: F_1,28_ = 4.47, *p* < 0.05; IL-6: F_1,28_ = 5.99, *p* < 0.05; TNF-α: F_1,28_ = 6.97, *p* < 0.05), and the CUS × BLLL formula interaction (IL-1β: F_1,28_ = 7.04, *p* < 0.05; IL-6: F_1,28_ = 4.25, *p* < 0.05; TNF-α: F_1,28_ = 4.62, *p* < 0.05) (Figures 2G–I). Post-hoc analysis revealed that 10-day BLLL formula treatment at a dose of 8 × 10^10^ CFU/kg reversed CUS-induced increases in the expression levels of IL-1β (Figure 2G), IL-6 (Figure 2H), and TNF-α (Figure 2I) in the hippocampus.

For the levels of IL-1β, IL-6, and TNF-α in the medial prefrontal cortex, the ANOVA showed significant effects for CUS exposure (IL-1β: F_1,28_ = 13.43, *p* < 0.01, IL-6: F_1,28_ = 24.14, *p* < 0.001, TNF-α: F_1,28_ = 11.43, *p* < 0.01), BLLL formula treatment (IL-1β: F_1,28_ = 8.39, *p* < 0.01; IL-6: F_1,28_ = 11.72, *p* < 0.01; TNF-α: F_1,28_ = 4.33, *p* < 0.05), and the CUS × BLLL formula interaction (IL-1β: F_1,28_ = 5.60, *p* < 0.05; IL-6: F_1,28_ = 7.79, *p* < 0.01; TNF-α: F_1,28_ = 8.28, *p* < 0.01) (Figures 2J,K). Post-hoc analysis revealed that 10-day BLLL formula treatment at a dose of 8 × 10^10^ CFU/kg could reverse CUS-induced increase in the expression levels of IL-1β (Figure 2J), IL-6 (Figure 2K), and TNF-α (Figure 2L) in the medial prefrontal cortex.

### BLLL formula reverses CUS-induced impairment of the anti-inflammatory responses in the hippocampal and medial prefrontal cortex in mice

**FIGURE 3:**
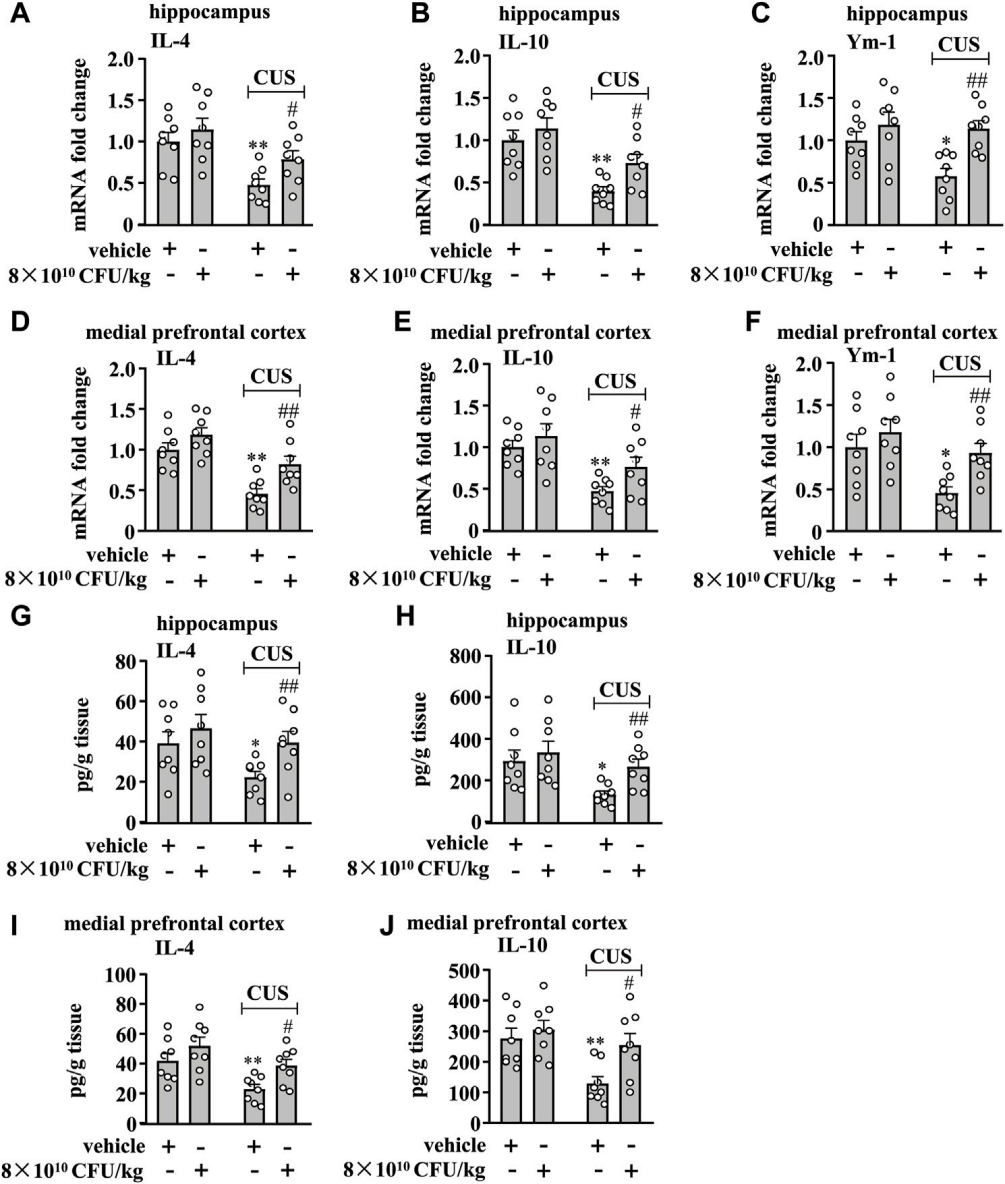
Effect of BLLL formula on anti-inflammatory responses in the brain in chronically stressed mice. **(A–C)** Quantitative analysis shows that 10 days of BLLL formula treatment (8 × 10^10^ CFU/kg) reversed CUS-induced decreases in the mRNA expression levels of IL-4 **(A)**, IL-10 **(B)**, and Ym-1 **(C)** in the hippocampus in mice (n = 8, *p < 0.05 or **p < 0.01 vs vehicle; #*p* < 0.05 or ##*p* < 0.01 vs. vehicle + CUS). **(D–F**) Quantitative analysis shows that 10 days of BLLL formula treatment (8 × 10^10^ CFU/kg) reversed CUS-induced decreases in the mRNA expression levels of IL-4 **(D)**, IL-10 **(E)**, and Ym-1 **(F)** in the medial prefrontal cortex in mice (n = 8, *p < 0.05 or *p < 0.01 vs vehicle; #*p* < 0.05 or ##*p* < 0.01 vs vehicle + CUS). **(G, H)** Quantitative analysis shows that 10 days of BLLL formula treatment (8 × 10^10^ CFU/kg) reversed CUS-induced decreases in levels of IL-4 **(G)** and IL-10 **(H)** in the hippocampus in mice (n = 8, *p < 0.05 vs vehicle; ##*p* < 0.01 vs vehicle + CUS). **(I, J)** Quantitative analysis shows that 10 days of BLLL formula treatment (8 × 10^10^ CFU/kg) reversed CUS-induced decreases in levels of IL-4 **(I)** and IL-10 **(J)** in the medial prefrontal cortex in mice (n = 8, **p < 0.01 vs vehicle; #*p* < 0.05 vs vehicle + CUS). Data are shown as mean ± SEM.

We also evaluated the effect of 10-day BLLL formula treatment on anti-inflammatory responses in the hippocampus and prefrontal cortex in chronically-stressed mice. A two-way ANOVA for levels of IL-4, IL-10, and Ym-1 mRNA in the hippocampus revealed significant effects for CUS exposure (IL-4 mRNA: F_1,28_ = 16.74, *p* < 0.001, IL-10 mRNA: F_1,28_ = 24.78, *p* < 0.001, Ym-1 mRNA: F_1,28_ = 4.46, *p* < 0.05) and BLLL formula treatment (IL-4 mRNA: F_1,28_ = 4.46, *p* < 0.05; IL-10 mRNA: F_1,28_ = 5.51, *p* < 0.05; Ym-1 mRNA: F_1,28_ = 11.33, *p* < 0.01), but not for CUS × BLLL formula interaction (IL-4 mRNA: F_1,28_ = 0.59, *p* = 0.45; IL-10 mRNA: F_1,28_ = 0.90, *p* = 0.35; Ym-1 mRNA: F_1,28_ = 2.87, *p* = 0.10) (Figures 3A–C). Post-hoc analysis revealed that 10-day BLLL formula treatment at the dose of 8 × 10^10^ CFU/kg reversed CUS-induced decrease in the expression levels of IL-4 (Figure 3A), IL-10 (Figure 3B), and Ym-1 (Figure 3C) mRNA in the hippocampus.

For the levels of IL-4, IL-10, and Ym-1 mRNA in the medial prefrontal cortex, the ANOVA showed significant effects for CUS exposure (IL-4 mRNA: F_1,28_ = 29.32, *p* < 0.001, IL-10 mRNA: F_1,28_ = 18.64, *p* < 0.001, Ym-1 mRNA: F_1,28_ = 9.98, *p* < 0.01) and BLLL formula treatment (IL-4 mRNA: F_1,28_ = 10.87, *p* < 0.01; IL-10 mRNA: F_1,28_ = 4.26, *p* < 0.05; Ym-1 mRNA: F_1,28_ = 6.78, *p* < 0.05), but not for the CUS × BLLL formula interaction (IL-4 mRNA: F_1,28_ = 1.18, *p* = 0.29; IL-10 mRNA: F_1,28_ = 0.59, *p* = 0.45; Ym-1 mRNA: F_1,28_ = 1.36, *p* = 0.25) (Figures 3D–F). Post-hoc analysis revealed that 10-day BLLL formula treatment at the dose of 8 × 10^10^ CFU/kg also reversed CUS-induced decrease in the expression levels of IL-4 (Figure 3D), IL-10 (Figure 3E), and Ym-1 (Figure 3F) mRNA in the medial prefrontal cortex.

We also evaluated the effect of 10-day BLLL formula treatment on CUS-induced decrease in IL-4 and IL-10 protein levels in the hippocampus and medial prefrontal cortex in mice. The two-way ANOVA for the levels of IL-4 and IL-10 in the hippocampus showed significant effects for CUS exposure (IL-4: F_1,28_ = 4.81, *p* < 0.05, IL-10: F_1,28_ = 7.55, *p* < 0.05) and BLLL formula treatment (IL-4: F_1,28_ = 5.26, *p* < 0.05; IL-10: F_1,28_ = 4.29, *p* < 0.05), but not for the CUS × BLLL formula interaction (IL-4: F_1,28_ = 0.79, *p* = 0.38; IL-10: F_1,28_ = 1.27, *p* = 0.27) (Figures 3G, H). The two-way ANOVA for the levels of IL-4 and IL-10 in the medial prefrontal cortex showed significant effects for CUS exposure (IL-4: F_1,28_ = 11.51, *p* < 0.01, IL-10: F_1,28_ = 9.96, *p* < 0.01) and BLLL formula treatment (IL-4: F_1,28_ = 7.54, *p* < 0.05; IL-10: F_1,28_ = 5.95, *p* < 0.05), but not for the CUS × BLLL formula interaction (IL-4: F_1,28_ = 0.36, *p* = 0.56; IL-10: F_1,28_ = 2.43, *p* = 0.13) (Figures 3I, J). Post-hoc analysis revealed that 10-day BLLL formula treatment at the dose of 8 × 10^10^ CFU/kg reversed CUS-induced decrease in levels of IL-4 (Figures 3G, I) and IL-10 (Figures 3H, J) in the hippocampus and medial prefrontal cortex.

### BLLL formula reverses CUS-induced changes in markers indicative of oxido-nitrosative stress in the hippocampus and medial prefrontal cortex

**FIGURE 4:**
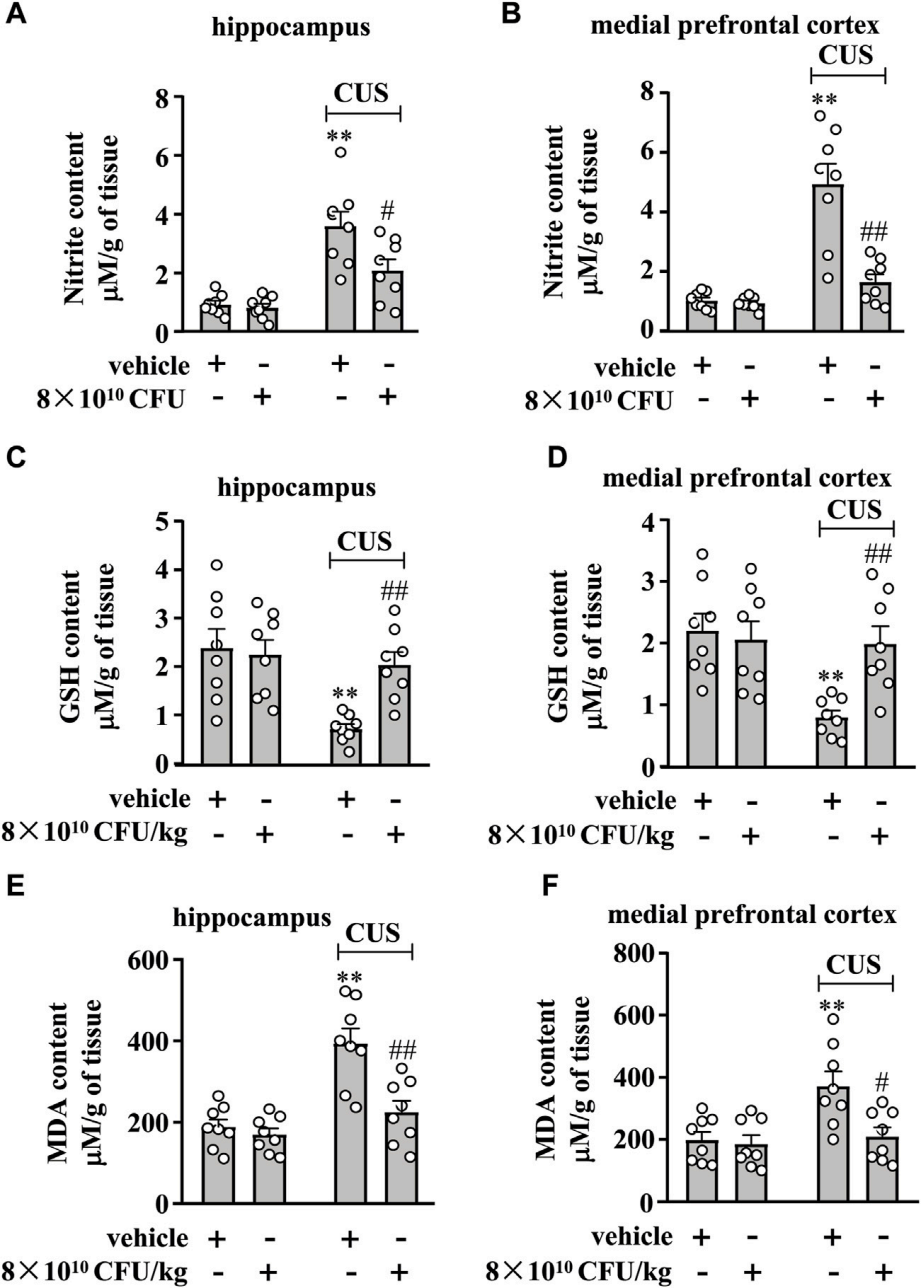
Effect of BLLL formula on CUS-induced changes in nitrite, GSH, and MDA content in the hippocampus and medial prefrontal cortex. **(A, B)** Quantitative analysis shows that BLLL formula treatment (8 × 10^10^ CFU/kg) reversed CUS-induced increases in levels of nitrite in the hippocampus **(A)** and medial prefrontal cortex **(B)** in mice (n = 8, **p < 0.01 vs vehicle; ##*p* < 0.01 vs vehicle + CUS). **(C, D)** Quantitative analysis shows that BLLL formula treatment (8 × 10^10^ CFU/kg) reversed CUS-induced decreases in contents of GSH in the hippocampus and medial prefrontal cortex in mice (n = 8, **p < 0.01 vs. vehicle; ##*p* < 0.01 vs. vehicle + CUS). **(E, F)** Quantitative analysis shows that BLLL formula treatment (8 × 10^10^ CFU/kg) reversed CUS-induced increases in contents of MDA in the hippocampus and medial prefrontal cortex in mice (n = 8, **p < 0.01 vs vehicle; #*p* < 0.05 or ##*p* < 0.01 vs vehicle + CUS). Data are shown as mean ± SEM.

We then examined the effect of the 10-day BLLL formula treatment on markers indicative of oxido-nitrosative stress in the hippocampus and medial prefrontal cortex in chronically stressed mice. A two-way ANOVA for nitrite levels in the hippocampus and medial prefrontal cortex showed significant effects for CUS exposure (hippocampus: F_1,28_ = 38.99, *p* < 0.001, cortex: F_1,28_ = 39.14, *p* < 0.001), BLLL formula treatment (hippocampus: F_1,28_ = 6.48, *p* < 0.05, cortex: F_1,28_ = 20.76, *p* < 0.001), and CUS × BLLL formula interaction (hippocampus: F_1,28_ = 4.91, *p* < 0.05, cortex: F_1,28_ = 18.77, *p* < 0.001) (Figures 4A, B). Post-hoc analysis revealed that 10-day BLLL formula treatment at a dose of 8 × 10^10^ CFU/kg reversed CUS-induced increase in nitrite levels in the hippocampus (Figure 4A) and medial prefrontal cortex (Figure 4B).

We also examined the effect of 10 days of BLLL treatment on GSH and MDA contents in the hippocampus and medial prefrontal cortex of chronically stressed mice. A two-way ANOVA for GSH content in the hippocampus and medial prefrontal cortex showed significant effects for CUS exposure (hippocampus: F_1,28_ = 10.94, *p* < 0.01, cortex: F_1,28_ = 8.69, *p* < 0.01), BLLL formula treatment (hippocampus: F_1,28_ = 4.33, *p* < 0.05, cortex: F_1,28_ = 4.40, *p* < 0.05), and the CUS × BLLL formula interaction (hippocampus: F_1,28_ = 6.68, *p* < 0.05, cortex: F_1,28_ = 7.19, *p* < 0.05) (Figures 4C, D). Post-hoc analysis revealed that 10-day BLLL formula treatment at a dose of 8 × 10^10^ CFU/kg reversed CUS-induced decrease in GSH content in the hippocampus (Figure 4C) and medial prefrontal cortex (Figure 4D).

For MDA content in the hippocampus and medial prefrontal cortex, the ANOVA showed significant effects for CUS exposure (hippocampus: F_1,28_ = 25.31, *p* < 0.001, cortex: F_1,28_ = 8.96, *p* < 0.01), BLLL formula treatment (hippocampus: F_1,28_ = 13.10, *p* < 0.01, cortex: F_1,28_ = 7.07, *p* < 0.05), and CUS × BLLL formula interaction (hippocampus: F_1,28_ = 8.37, *p* < 0.01, cortex: F_1,28_ = 5.05, *p* < 0.05) (Figures 4E, F). Post-hoc analysis revealed that 10-day BLLL formula treatment at a dose of 8 × 10^10^ CFU/kg reversed the CUS-induced increase in MDA content in the hippocampus (Figure 4E) and medial prefrontal cortex (Figure 4F).

### BLLL formula reverses CUS-induced decrease in BDNF levels in the hippocampal and medial prefrontal cortex

**FIGURE 5:**
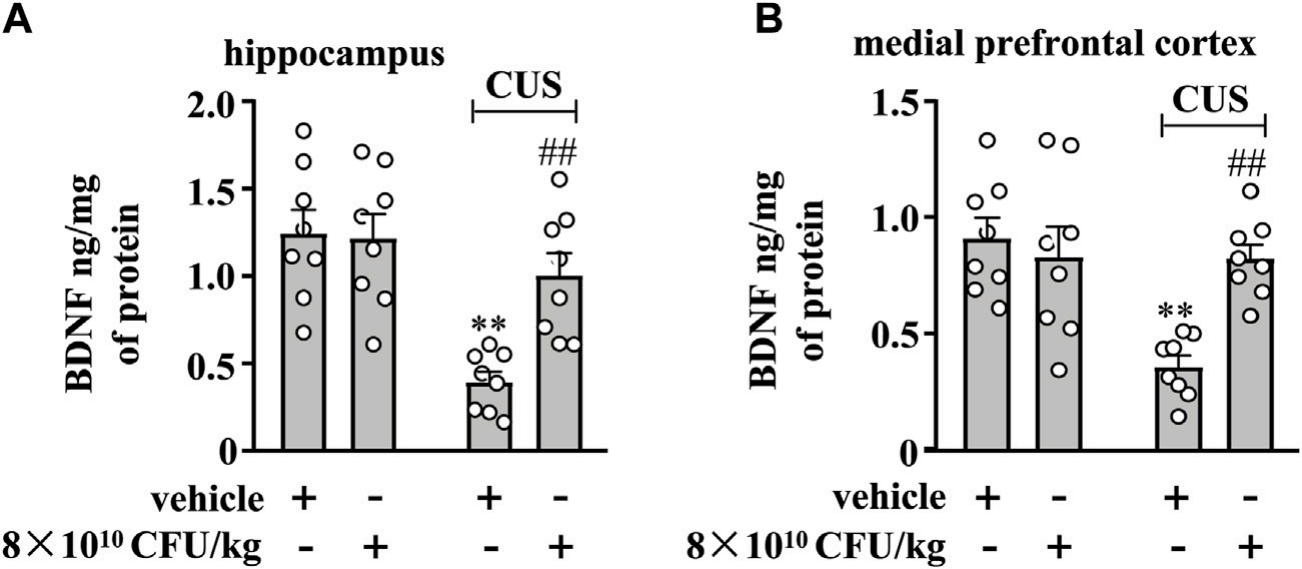
Effect of BLLL formula on CUS-induced decreases in levels of BDNF in the hippocampus and medial prefrontal cortex. **(A, B)** Quantitative analysis shows that BLLL formula treatment (8 × 10^10^ CFU/kg) reversed CUS-induced decreases in levels of BDNF protein in the hippocampus **(A)** and medial prefrontal cortex **(B)** in mice (n = 8, **p < 0.01 vs. vehicle; ##*p* < 0.01 vs. vehicle + CUS). Data are shown as mean ± SEM.

Finally, we evaluated the effect of 10-day BLLL formula treatment on BDNF content in the hippocampus and medial prefrontal cortex in chronically-stressed mice. A two-way ANOVA for the change of BDNF content in the hippocampus and medial prefrontal cortex showed significant effects for CUS exposure (hippocampus: F_1,28_ = 19.66, *p* < 0.001, cortex: F_1,28_ = 10.75, *p* < 0.01), BLLL formula treatment (hippocampus: F_1,28_ = 5.99, *p* < 0.01, cortex: F_1,28_ = 5.11, *p* < 0.05), and CUS × BLLL formula interaction (hippocampus: F_1,28_ = 7.11, *p* < 0.05, cortex: F_1,28_ = 10.04, *p* < 0.01) (Figures 5A, B). Post-hoc analysis revealed that 10-day BLLL formula treatment at a dose of 8 × 10^10^ CFU/kg reversed CUS-induced decrease in BDNF content in the hippocampus (Figure 5A) and medial prefrontal cortex (Figure 5B).

## Discussion

One of the main contributions of the present study was the identification of an apparent antidepressant-like effect of a new probiotic formula consisting of four types of probiotics *B. breve*, *L. plantarum*, *L. paracasei*, and *L. helveticus* (called BLLL formula) in mice subjected to chronic stress in behavioral tests such as TSF, FST, and SPT. The antidepressant-like effect of BLLL was significant when administered at 8 × 10^10^ CFU/kg for 10 days. The reduction in immobility in TST and FST in the BLLL-treated mice was not attributed to changes in their endogenous spontaneous locomotor activity, indicating that the formula indeed has an antidepressant-like effect. This hypothesis was further supported by the finding that continuous administration of the BLLL formula for 10 days reversed the CUS-induced reduction in sucrose intake in mice. As in-depth studies on the regulatory effects of probiotics on the nervous system are ongoing worldwide, supplementing probiotics with neuroprotective effects is now considered a potential strategy for the treatment of nervous system disorders, including depression (Aghamohammad et al., 2023; Varesi et al., 2023). Our results represent a potential option for this purpose and may be of interest to those aiming to develop new antidepressants based on probiotics. It is worth noting that the antidepressant-like effects of the individual components of BLLL have already been described in multiple prior studies (Maehata et al., 2019; Tian et al., 2019; Tian et al., 2020; Partrick et al., 2021; Xu et al., 2022; Zhu R. et al., 2023; Kwon et al., 2023; Ma et al., 2024). As the efficacy of probiotics is both strain-specific and outcome-specific (McFarland et al., 2021), the supplementation of a multi-strain probiotic formula may have additive or synergistic effects that require thorough investigation in future studies.

In recent decades, there have been numerous reports on the therapeutic effects of probiotics on depressive behavior in both animals and humans (Huang et al., 2016; Park et al., 2018; Kosuge et al., 2021; Tian et al., 2022). For example, *B. breve* has been shown to have significant antidepressant-like effects in chronically stressed C57BL/6J mice (Tian et al., 2020; Sushma et al., 2023). The administration of *L. paracasei* and *L. plantarum* has been found to prevent stress-induced anxiety- and depression-like behaviors in Swiss mice (Stenman et al., 2020). *Lactobacillus rhamnosus*, combined with *L. helveticus*, has been reported to alleviate anxiety- and depression-like behaviors in B- and T-cell-deficient Rag1^−/−^ mice (Smith et al., 2014). All of these studies begin with the administration of probiotics before or at the onset of stress and continue for four or 5 weeks until the end of chronic stress [63–65]. However, it is worth noting that it is almost impossible to administer medication or probiotics before or at the onset of stress. Our study holds greater clinical significance because the probiotic formula was administered after stress stimulation was discontinued. In addition, while numerous prior studies focus on a single strain, our investigation explored the impact of a mixture of four stains of probiotics on depression-like behaviors in animals. Although the mechanisms underlying the antidepressant-like effect of probiotics on depression are still largely unclear, many studies have reported that metabolites produced by probiotics in the gut may mediate these effects. According to the dose-dependent theory, it is reasonable to assume that the efficacy of the antidepressant-like effects of probiotics could be influenced by the dosage used. The mixture of different strains of probiotics may enhance the antidepressant-like effects of probiotics by producing more anti-inflammatory and anti-oxidative agents. These hypotheses should be investigated in future studies.

The development of new antidepressants remains a challenge due to the unclear biological mechanisms underlying depression. One possible reason for this could be that too much attention is paid to neuronal dysfunction. In recent years, numerous studies have investigated the connection between depression and neuroinflammation as well as oxido-nitrosative stress (Sulakhiya et al., 2014; Yang et al., 2017; Dantzer et al., 2018; Himmerich et al., 2019; Nowak et al., 2019; Osimo et al., 2020; Cosma et al., 2021; Perry et al., 2021). Chronic stress in animals, such as CUS, chronic social defeat stress, and chronic restraint stress, can lead to an increase in pro-inflammatory cytokines in the brain, like IL-6, IL-1β, and TNF-α. These cytokines could trigger microglial hyper-activation, neuroinflammation, and impairment of the BDNF pathway, ultimately leading to neuronal damage and the development of depressive symptoms that can be assessed by behavioral experiments such as TST, FST, and SPT. Furthermore, the direct infusion of pro-inflammatory cytokines such as IL-1β and TNF-α into the brain has been shown to induce depression-like behaviors in rodents (Anisman et al., 2008; Hayley et al., 2013). In humans, administrating pro-inflammatory substance LPS can promote an increase of pro-inflammatory cytokines in serum, thereby triggering illness and depression-like behaviors (Maes et al., 2008; Benson et al., 2017). Here, we found that 35-day CUS exposure elicited a significant increase in pro-inflammatory cytokine levels (such as IL-6, IL-1β, and TNF-α) in the hippocampus and medial prefrontal cortex of mice while also reducing the levels of anti-inflammatory factors (such as IL-4, IL-10, and Ym-1) in these brain regions. BLLL administration suppressed the CUS-induced increase in IL-6, IL-1β, and TNF-α and the CUS-induced decrease in IL-4, IL-10, and Ym-1 in the hippocampus and medial prefrontal cortex. IL-4 and IL-10 are typical factors with anti-inflammatory effects. They can transform inflammatory cells such as macrophages and microglia from a pro-inflammatory state to an anti-inflammatory state, which could be beneficial for tissue remodeling, repair, and neuroprotection. Ym1, a secreted and self-crystallizing protein, is encoded by a gene that was cloned from mouse peritoneal-activated macrophages (Chang et al., 2001) and is also expressed in microglia (Li et al., 2024), glandular gastric epithelium (Ward et al., 2001) or neutrophils (Harbord et al., 2002). As an inflammation-responsive protein (Raes et al., 2002), increased Ym-1 expression can help limit the progression of inflammation and can be further enhanced by interleukin four-induced gene-1 (Yue et al., 2015). Under BLLL treatment, the rise in Ym-1 in CUS-exposed mice could shift the neuroinflammatory responses in the brain towards an anti-inflammatory phenotype, thereby inducing an antidepressant-like effect. The relationship between Ym-1 and IL-4 or IL-10 should be carefully investigated in future studies.

Our results also showed that CUS caused a significant reduction in BDNF levels in the hippocampus and medial prefrontal cortex and that these effects of CUS were reversed by administration of the BLLL formula. This finding is in strong agreement with previous reports confirming the link between neuroinflammation and the BDNF signaling pathway (Calabrese et al., 2014; Yang et al., 2017). Considering that pro-inflammatory cytokines have been widely described as mediators of immobile and anhedonic behavior in TST, FST, and SPT, we concluded that administration of BLLL could attenuate CUS-induced depression-like behavior in mice in part by suppressing neuroinflammation that accompanied the restoration of BDNF expression. However, further investigation is required to understand how the BLLL formula affects the production of pro-inflammatory cytokines and BDNF in the brain. We also can not overlook the possibility that the BLLL formula could influence the expression of pro-inflammatory cytokines and BDNF in the brain in an independent manner. Additionally, since microglia can also produce BDNF, it needs to clarify whether the increased BDNF levels in the brain of CUS mice after administration of BLLL originate from microglia. These questions should be addressed in future studies.

Aside from neuroinflammation, oxidative and nitrosative stress may also play an important role in depression (Sulakhiya et al., 2014; Yang et al., 2017). It has been reported that the over-activated microglia and innate immune system increase oxidative and nitrosative stress (Morris et al., 2018; Bittencourt et al., 2022). Direct administration of pro-inflammatory cytokines such as TNF-α and IL-6 to mice also increases reactive oxygen species (ROS) by increasing the production of NO (Madrigal et al., 2002). The increased NO, along with ROS, causes oxido-nitrosative stress, which leads to neurotoxic effects through various mechanisms, such as the reduction of BDNF expressions and monoamine levels (Kasala et al., 2014; Amiry et al., 2023). This opinion was supported by our data, as some markers that reflect the status of oxidative and nitrosative stress, such as MDA, GSH, and nitrite, were significantly altered by CUS exposure. Increased MDA and nitrite levels reflect higher oxidative and nitrosative status, while decreased GSH levels indicate lower antioxidant status (Rongzhu et al., 2009; Borges et al., 2015). Reversing the deleterious changes in these markers would be beneficial for the treatment of major depression. Our studies showed that administration of the BLLL formula significantly reversed the CUS-induced increase in MDA and nitrite levels and the CUS-induced decrease in GSH levels in the hippocampus and prefrontal cortex. These findings suggest that the suppression of oxido-nitrosative stress may be a key mechanism for the reversal of depression-like behavior using the BLLL formula. Whether the antioxidant effect of the BLLL formula is related to its anti-neuroinflammatory effect should be investigated in future studies.

It is worth noting that the same dose and duration of BLLL treatment has no effect on the behavior of non-stressed control mice in the TST, FST, and SPT, as well as on the levels of IL-1β, IL-6, TNF-α, IL-4, IL-10, Ym-1, nitrite, MDA, GSH, BDNF in the hippocampus and medial prefrontal cortex of the non-stressed control mice. It is interesting to consider why BLLL only affects the above parameters in chronically stressed mice. Since chronic stress exposure is a pathological stimulation, we hypothesize that 35-day CUS stimulation may disrupt the homeostasis of some targets that mediate the production of pro-inflammatory cytokines and the progression of oxidative and nitrosative stress, such as nuclear factor-kappa B (NF-κB) and nuclear factor erythroid-derived 2-like 2 (Nrf2), thereby promoting the development of depression-like behaviors. Numerous studies have reported that targets such as NF-κB and Nrf2 could be affected by different types of probiotics in animal models of depression (Gao et al., 2021; Zhu L. et al., 2023; Fatima et al., 2023; Rezaie et al., 2024). In future studies, we will further investigate how exactly the progression of neuroinflammation and oxidative stress in the brain is affected under chronic stress conditions.

There are some limitations to this study that need to be acknowledged. First, we only investigated the antidepressant-like effect of BLLL in male mice but not in female mice. This is very important because female and male mice usually display different behavioral and biological responses to stress. For instance, in the helplessness model, female animals can learn to escape from stressful tasks and, therefore, do not exhibit helplessness behavior (Heinsbroek et al., 1991). In some other studies, female animals have shown a delayed return to baseline corticosterone levels after abnormal activation of the hypothalamic-pituitary-adrenal axis (Viau et al., 2005; Dudek et al., 2021). In addition, female animals can demonstrate a more efficient anti-inflammatory adaptation to chronic stress (Bollinger et al., 2020). In future studies, we will investigate whether the antidepressant-like effect of BLLL also applies to female animals. Second, in the present study, we only examined the parameters reflecting neuroinflammation and oxidative stress in the hippocampus and medial prefrontal cortex to illustrate the mechanisms underlying the antidepressant-like effect of BLLL. However, there are many other brain regions, such as the amygdala, nucleus accumbens, and hypothalamus, that respond to neuroinflammation and oxidative stress and may be associated with depression (Wang J. et al., 2022; Xu et al., 2023; Zhang et al., 2023). Therefore, future studies should investigate the cellular and regional basis for the antidepressant-like effects of BLLL. Furthermore, the BLLL probiotics supplement used here can directly influence the gut microbiota. To this end, future studies should also explore other mechanisms, besides anti-neuroinflammation and anti-oxidative stress, that are related to gut dysbiosis and the brain-gut axis, as well as the absorption of the metabolites associated with BLLL administration.

## Conclusion

**FIGURE 6:**
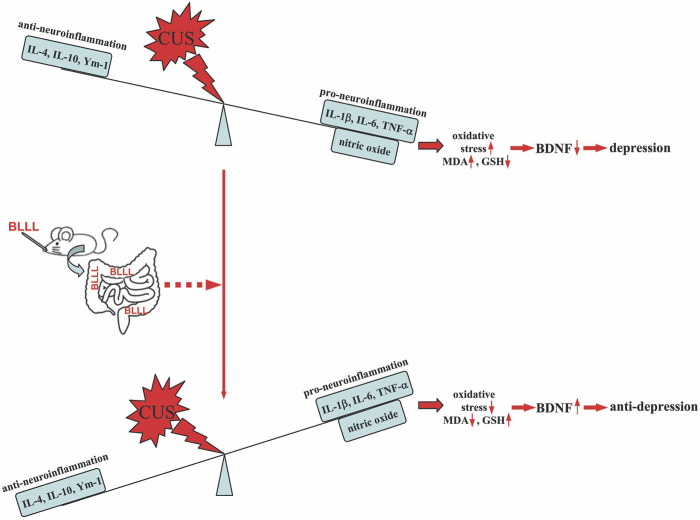
A schematic illustration showing that oral administration of BLLL reverses chronic stress-induced depression-like behavior in mice by converting the neuroinflammatory responses to an anti-inflammatory state, as indicated by the decrease in the levels of IL-1β, IL-6, and TNF-α and the increase in the levels of IL-4, IL-10, and Ym-1 in the brain. This would subsequently rebalance the levels of MDA, GSH, and BDNF in the brains of chronically stressed mice.

Our results show that repeated administration of the BLLL formula, a newly developed probiotic consisting of *B. breve*, *L. plantarum*, *L. paracasei*, and *L. helveticus*, can reverse chronic stress-induced depression-like behavior in mice, which is achieved by reducing neuroinflammation and oxido-nitrosative stress, and by increasing BDNF levels in the brain (Figure 6). Given the close association between gut microbiota dysfunction and the development of depression (Huang et al., 2016; Park et al., 2018; Kosuge et al., 2021; Tian et al., 2022), as well as the individual components of the BLLL formula having demonstrated apparent antidepressant-like effects in patients with depression (Rode et al., 2022; Duarte et al., 2023; Önning et al., 2023; Sarkawi et al., 2024), our findings suggest that the BLLL formula used in the present study has the potential to be developed as a new drug for the treatment of depression in the clinic. However, before this goal can be achieved, further studies should be conducted to clarify the effects, metabolism, conversion, stability, and safety of the BLLL formula in humans.

## Data Availability

The raw data supporting the conclusions of this article will be made available by the authors, without undue reservation.
